# Oral Health in Adult Patients Receiving Palliative Care: A Mixed Method Study

**DOI:** 10.1093/geroni/igab046.1597

**Published:** 2021-12-17

**Authors:** Xi Chen, Violet D'Souza, Timothy Thomsen, Stephanie Gilbertson-White, Jirakate Madiloggovit, Chandler Pendleton, Xianjin Xie, Arshi Munjal

**Affiliations:** 1 University of Iowa, Iowa City, Iowa, United States; 2 University of Toronto, Toronto, Ontario, Canada; 3 University of Iowa Roy J. and Lucille A. Carver College of Medicine, Iowa City, Iowa, United States; 4 University of Iowa College of Nursing, Iowa City, Iowa, United States; 5 University of Iowa College of Dentistry, Iowa City, Iowa, United States

## Abstract

Oral disease is highly prevalent in persons receiving palliative care (PRPC). Yet, little is known about how PRPC perceive their oral health status and related treatment needs. Forty-nine PRPC were recruited. They first completed a structured oral symptom review, followed by an oral exam. A nested sample of 11 participants also completed an in-depth interview querying their perceived oral health concerns and related treatment needs. Quantitative and qualitative data was analyzed and integrated for interpretation. Eighty-six percent of participants reported at least one oral symptom, including dry mouth (83.7%), a pain-related symptom (40.8%), or oral function difficulties (51.0%). About 40% of participants reported compromised quality of life due to oral conditions; however, the perceived impacts and treatment needs were modest. Oral disease was highly prevalent in PRPC, yet its overall impact was modest. Except for painful symptoms, most participants reported limited desire to seek treatment for oral health conditions.

